# Metabolomic Salivary Signature of Pediatric Obesity Related Liver Disease and Metabolic Syndrome

**DOI:** 10.3390/nu11020274

**Published:** 2019-01-26

**Authors:** Jacopo Troisi, Federica Belmonte, Antonella Bisogno, Luca Pierri, Angelo Colucci, Giovanni Scala, Pierpaolo Cavallo, Claudia Mandato, Antonella Di Nuzzi, Laura Di Michele, Anna Pia Delli Bovi, Salvatore Guercio Nuzio, Pietro Vajro

**Affiliations:** 1 Department of Medicine and Surgery and Dentistry, “Scuola Medica Salernitana”, Pediatrics Section University of Salerno, 84081 Baronissi (Salerno), Italy; fecu91@gmail.com (F.B.); a.bisogno91@gmail.com (A.B.); luca.pierri@hotmail.com (L.P.); angelocolucci2@gmail.com (A.C.); antonelladinuzzi@gmail.com (A.D.N.); lauradimichele05091993@gmail.com (L.D.M.); delliboviannapia@gmail.com (A.P.D.B.); sguercio.nuzio@gmail.com (S.G.N.); pvajro@unisa.it (P.V.); 2 Theoreo srl, Via degli Ulivi 3, 84090 Montecorvino Pugliano (SA), Italy; 3 European Biomedical Research Institute of Salerno (EBRIS), Via S. de Renzi, 3, 84125 Salerno, Italy; 4 Hosmotic srl, Via R. Bosco 178, 80069 Vico Equense (NA), Italy; scala@hosmotic.com; 5 Department of Physics, University of Salerno, 84084 Fisciano (Salerno), Italy; pcavallo@unisa.it; 6 Department of Pediatrics, Children’s Hospital Santobono-Pausilipon, 80129 Naples, Italy; cla.mandato@gmail.com; 7 European Laboratory of Food Induced Intestinal Disease (ELFID), University of Naples Federico II, 80100 Naples, Italy

**Keywords:** pediatric obesity, nonalcoholic fatty liver disease, metabolic syndrome, saliva, metabolomics, gas-chromatography mass spectrometry

## Abstract

Pediatric obesity-related metabolic syndrome (MetS) and nonalcoholic fatty liver disease (NAFLD) are increasingly frequent conditions with a still-elusive diagnosis and low-efficacy treatment and monitoring options. In this study, we investigated the salivary metabolomic signature, which has been uncharacterized to date. In this pilot-nested case-control study over a transversal design, 41 subjects (23 obese patients and 18 normal weight (NW) healthy controls), characterized based on medical history, clinical, anthropometric, and laboratory data, were recruited. Liver involvement, defined according to ultrasonographic liver brightness, allowed for the allocation of the patients into four groups: obese with hepatic steatosis ([St+], *n* = 15) and without hepatic steatosis ([St–], *n* = 8), and with (*n* = 10) and without (*n* = 13) MetS. A partial least squares discriminant analysis (PLS-DA) model was devised to classify the patients’ classes based on their salivary metabolomic signature. Pediatric obesity and its related liver disease and metabolic syndrome appear to have distinct salivary metabolomic signatures. The difference is notable in metabolites involved in energy, amino and organic acid metabolism, as well as in intestinal bacteria metabolism, possibly reflecting diet, fatty acid synthase pathways, and the strict interaction between microbiota and intestinal mucins. This information expands the current understanding of NAFLD pathogenesis, potentially translating into better targeted monitoring and/or treatment strategies in the future.

## 1. Introduction

The incidence of obesity and its related conditions, including metabolic syndrome (MetS) and non-alcoholic fatty liver disease (NAFLD), has dramatically increased worldwide in all age groups including pediatrics [1]. Pediatric obesity definitely is an early risk factor for adult morbidity and mortality [2,3]. Due to the existence of a well-established tracking phenomenon, the early detection and treatment of MetS and fatty liver in childhood represents a valuable tool to prevent further health complications and to minimize the global socioeconomic burden of hepato-metabolic and cardiovascular obesity-associated complications in adulthood [4]. Although the exact definition of MetS is still debated regarding the pediatric population, most researchers agree (a) that it includes hypertension, hyperglycemia, dyslipidemia together with visceral obesity, and (b) that NAFLD has to be considered its hepatic component.

Metabolomics has recently started to pave the way to a better pathomechanistic understanding of these hepatometabolic complications, leading to a more efficient diagnosis and better therapeutic approaches. In this regard, studies have shown that high urinary/blood levels of aromatic (AAA) ± branched chain (BCAA) amino acids are known to be associated with insulin resistance (IR) and the risk of obesity-related MetS [5,6,7,8].

Lipid metabolism, tyrosine [9], alanine and the urea cycle [5], acylcarnitine catabolism ± changes in nucleotides, lysolipids, and inflammation markers [10], and several other components [11,12,13] also appear to be implicated in obesity and its related disorders. 

We have recently shown a complex network of urinary molecules prevalently represented by intestinally-derived bacterial products [14] which are correlated with the clinical phenotype and can differentiate between normal weight and obese children, distinguishing between those with and without liver involvement, based also on the characteristics of their gut-liver axis (GLA) function [15].

To identify an even more easily accessible and readily obtained biofluid for possible minimally invasive disease recognition [16], few studies have shown saliva suitability for investigations of individual metabolites of oxidative stress in obesity [17] and obesity-related MetS/NAFLD [4,18]. We showed that salivary testing of uric acid, glucose, insulin and HOMA together with selected anthropometric parameters may help to identify noninvasively obese children with hepatic steatosis and/or having MetS components [4]. However, salivary metabolomics studies in this respect are lacking. 

Based on these and a few other urine-and/or plasma-based metabolomic studies of pediatric obesity and MetS [15,19,20,21], we hypothesized that differences in the metabolite profiling of lean and obese children with and without NAFLD/MetS might also be evident in saliva, which might be ideal to screen noninvasively obese children at a higher risk of hepatometabolic complications. Prospectively, better delineation of individual or clusters of specific metabolites could serve as diagnostic biomarkers to be further investigated in future studies appraising even early stages of these comorbidities.

## 2. Materials and Methods 

### 2.1. Population and Study Design

Among 46 consecutive subjects (aged 7–15 years) seen at our obesity clinic or planned for only minor surgery, 41 with verified good oral health and not taking medications were enrolled in a nested case-control study over a transversal design. Eighteen had a normal weight (NW; body mass index (BMI) < 85th percentile) and 23 were obese (BMI > 95th percentile). The patients were characterized based on clinical, anthropometric (blood pressure, BMI, waist circumference (WC), and neck circumference (NC)), laboratory (serum alanine aminotransferase (ALT), aspartate aminotransferase (AST), total and high-density lipoprotein (HDL) cholesterol, triglycerides, uric acid (UA), glucose, and insulin) parameters. An ultrasound (US) was used to determine the presence [St+] or absence [St–] of hepatic steatosis [22,23]. Blood tests were performed using a standard laboratory analyzer (Abbott Diagnostics, Santa Clara, CA, USA). 

ALT upper normal values referred either to the customary normal range cut-off value of 40 IU/L or more precise SAFETY study cut-off pediatric values of 25.8 and 22.0 IU/L for boys and girls, respectively [24].

Patients with hepatic steatosis and/or transaminases >1.5 times the upper customary normal values were screened for celiac disease, Wilson disease, autoimmune hepatitis, and major and minor hepatotropic viruses [25]. According to the International Diabetes Foundation (IDF), MetS was defined as the presence of at least three of the following parameters: WC >95th percentile; triglycerides >150 mg/dL; blood glucose >100 mg/dL; systolic blood pressure (SBP) >95th percentile; and HDL cholesterol <40 mg/dL [26]. 

### 2.2. Saliva Samples

Each subject was asked to refrain from eating, drinking and brush tooting procedures for at least 1 h before saliva collection. Then he/she underwent a morning, whole saliva sampling using a saliva cotton roll commercial collection device (Salivette^®^; Sarstedt, Nümbrecht, Germany). As recommended by the manufacturer, to stimulate salivation patients, patients were asked to roll and gently chew the cotton swab in their mouth for 60–90 s. Then the swab was spitted in the collection tube of the kit and centrifuged within 1 h at 2000× *g* for 2 min. The collected clear, fluid saliva sample was aliquoted without any further processing and frozen at −80 °C until samples’ analysis, as previously described [4].

### 2.3. Ethical Approval

The study complied with the terms of the Declaration of Helsinki of 1975 (as revised in 2013) [27] for the investigation of human subjects, with written informed consent from patients and their families. All participants agreed to participate in this study and contribute saliva samples for metabolomic analysis. All samples were collected in accordance with the ethical guidelines mandated by and approved by our institutional Health Research Ethics Board. The study protocol was approved by the Ethics Review Committee of the University Hospital S. Giovanni di Dio e Ruggi d’Aragona of Salerno (Prot. No 18.02.2013/98).

### 2.4. Untargeted Metabolomics Analysis

#### 2.4.1. Metabolites Extraction and Derivatization

Metabolome extraction, purification and derivatization were carried out using the MetboPrep GC kit (Theoreo srl, Montecorvino Pugliano (SA), Italy) according to the manufacturer’s instructions.

#### 2.4.2. GC-MS Analysis

GC-MS analysis was performed on the derivatized extracted metabolome according to Troisi et al. [15] with a few minor changes. Briefly, 2 µL of the sample solution was injected into the GC-MS system (GC-2010 Plus gas chromatograph coupled to a 2010 Plus single quadrupole mass spectrometer; Shimadzu Corp., Kyoto, Japan) equipped with a 30-m, 0.25-mm ID CP-Sil 8 CB fused silica capillary GC column with 1.00-µm film thickness from Agilent (Agilent, J&W Scientific, Folsom, CA, USA), using He as a carrier gas. The initial oven temperature of 100 °C was maintained for 1 min and then raised by 6 °C/min to 320 °C with a further 2.33 min of hold time. The gas flow was set to obtain a constant linear velocity of 39 cm/s, and injections were performed in the splitless mode. The mass spectrometer was operated in electron impact (70 eV) in the full-scan mode in the interval of 35–600 m/z with a scan velocity of 3333 amu/s and a solvent cut-off time of 4.5 min. The complete GC analysis duration was 40 min. Untargeted metabolites were identified by comparing the mass spectrum of each peak with the NIST library collection (NIST, Gaithersburg, MD, USA).

#### 2.4.3. Metabolites Identification 

Of the over 240 signals per sample produced by GC-MS analysis, only 222 were investigated further because they were consistently found in at least 85% of samples.

To identify metabolites under the peaks, the Kovats’ index [28] difference max tolerance was set at 10, while the minimum matching for the NIST library search was set at 85%. The results were summarized in a comma-separate matrix file and loaded in the appropriate software for statistical manipulation. The chromatographic data for PLS-DA analysis were tabulated with one sample per row and one variable (metabolite) per column. The normalization procedures consisted of data transformation and scaling. Data transformation was made by generalized log transformation and data scaling by autoscaling (mean-centered and divided by standard deviation of each variable) [29]. Relevant metabolites selected using statistical analysis were further confirmed with an analytical standard purchased from Sigma-Aldrich (Milan, Italy) as indicated in the Metabolomic Standard Initiative reports [30].

### 2.5. Statistical Analysis 

#### 2.5.1. Demographical and Clinical Data

Statistical analysis was performed using Statistica software (StatSoft, Tulsa, OK, USA) and Minitab (Minitab Inc., State College, PA, USA). The normal distribution of data was verified using the Shapiro–Wilks test. Because the data were normally distributed, we used one-way ANOVA with Tukey’s post-hoc test for intergroup comparisons. A result with *p* < 0.05 was considered statistically significant.

#### 2.5.2. Metabolomics Univariate Data Analysis

Metabolite concentration differences among the classes (NW, OB[St+] and OB[St−]) were evaluated in terms of fold change (FC) and *p*-value (assessed using Student’s *t*-test because the metabolite amount was previously normalized).

The volcano plot representation was used to encounter both criteria. Metabolites with high FC (>1 or <−1) and lower *p*-value (<0.05) were selected as the most relevant.

#### 2.5.3. Metabolomic Multivariate Data Analysis

Partial least squares discriminant analysis (PLS-DA) was performed on the internal standard peak area [31] normalized chromatogram using R (Foundation for Statistical Computing, Vienna, Austria). Mean centering and unit variance scaling were applied for all analyses. Class separation was archived by PLS-DA, which is a supervised method that uses multivariate regression techniques to extract, via linear combinations of original variables (X), the information that can predict class membership (Y). PLS regression was performed using the *plsr* function included in the R pls package [32]. Classification and cross-validation were performed using the wrapper function included in the caret package [33]. A permutation test was performed to assess the significance of class discrimination. In each permutation, a PLS-DA model was built between the data (X) and permuted class labels (Y) using the optimal number of components determined by cross validation for the model based on the original class assignment. Two types of test statistics were used to measure class discrimination. The first is based on prediction accuracy during training. The second used separation distance based on the ratio of the between groups sum of the squares and the within group sum of squares (B/W-ratio). If the observed test statistics were part of the distribution based on the permuted class assignments, class discrimination cannot be considered significant from a statistical point of view [34]. Variable importance in projection (VIP) scores were calculated for each component. A VIP score is a weighted sum of squares of the PLS loadings, considering the amount of explained Y-variation in each dimension.

The metabolic pathway was constructed using the MetScape application [35] of the software Cytoscape [36].

## 3. Results

The demographic and clinical laboratory characteristics of the case and control subjects are reported in Table 1. None of the NW controls had either biochemical or US hepato-metabolic abnormalities. 

More than 50% of obese children (*n* = 15) had ultrasonographic (US) signs of NAFLD and hypertransaminasemia not due to the most common causes of liver diseases, as well as significantly higher values of systolic blood pressure (127 ± 9 vs. 96 ± 11 mm Hg, *p* = 0.0003) and glycemia (88.6 ± 10.4 vs. 83.2 ± 6.6 mg/dL, *p* = 0.002) compared with NW subjects. Twenty-one patients had no component of MetS, 7 had at least one component, 10 had two or three components, and only 3 had more than three components (Table 2).

**Figure 1:**
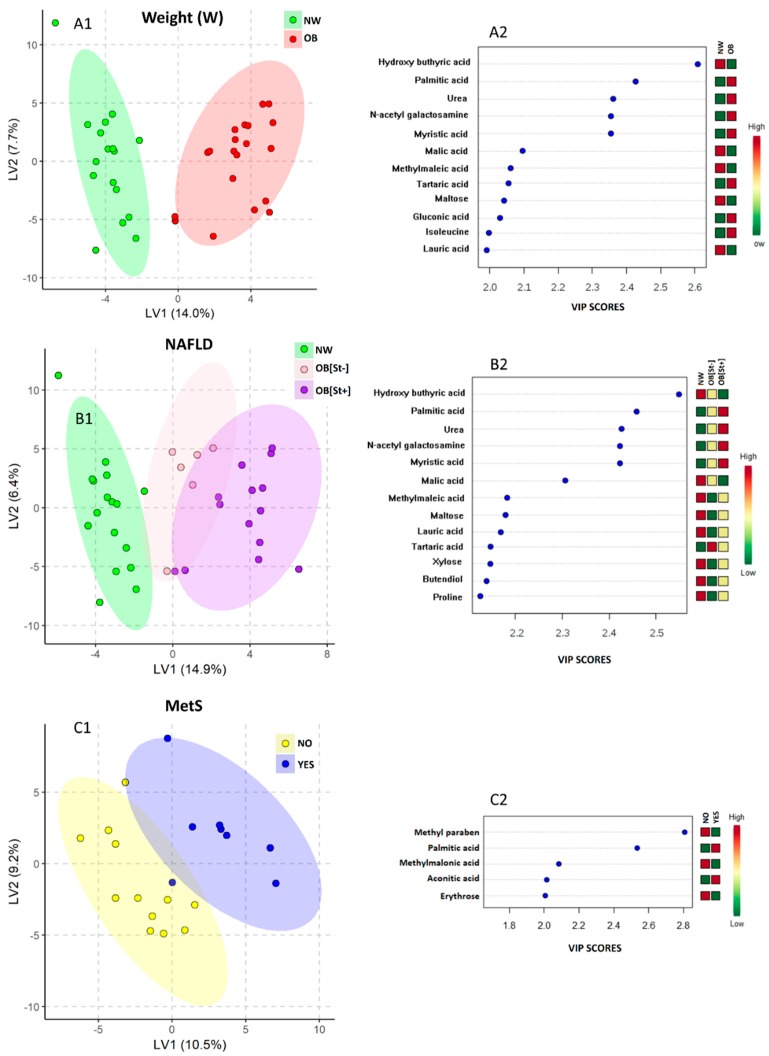
Partial least square discriminant analysis (PLS-DA) models to discriminate children according to Body Mass Index (BMI) (**A1**) and Non Alcoholic Fatty Liver Disease (NAFLD) (**B1**), as unique parameters investigated. The explained variance of each component is shown in parenthesis on the corresponding axis. In panel **A1**, the green ellipse contains normal weight children, while the red one contains the obese children. In panel **B1**, the purple circles represent the obese children with NAFLD (OB[St+]), the pink circles represent obese children without NAFLD (OB[St−]), while green circles represent the normal weight controls (NW). In panel **C1**, the blue circles represent the children with a diagnosis of metabolic syndrome (MetS), while the yellow ones represent the children without MetS diagnosis. The first 12, 13 and 5 variables important in projection (VIP) identified by the corresponding PLS-DA are shown in Panels **A2**, **B2** and **C2** respectively. The number of VIPs was established by setting the VIP-score ≥ 2 as a cut off value. In all cases, the colored boxes on the right indicate the relative amount of the corresponding metabolite in each group under study.

As shown in Figure 1, the PLS-DA score plots clearly differentiated between obese (OB) and normal weight (NW) children (Figure 1A1) and between OB with and without steatosis and NW controls (Figure 1B1). Twelve and 13 metabolites with a VIP-score > 2 separated NW/OB and NW/OB[St+]/OB[St–], respectively (Figure 1A2,B2). A third PLS-DA model (Figure 1C1) separated children according to MetS via five metabolites that had a VIP-score >2 (Figure 1C2).

As shown in Figure 1 and Table 3, compared with NW subjects, the saliva of obese children had higher levels of palmitic acid, myristic acid, urea, *N*-acetyl galactosamine, maltose, gluconic acid and isoleucine and lower levels of hydroxy butyric acid and malic acid, which were prevalent in those without steatosis and lauric acid, maltose and methyl maleic acid, which were prevalent in those with steatosis.

The volcano plot representation and histogram of the metabolites selected using volcano plot analysis (FC > 1 or < −1, *p* < 0.05) of the OB patients compared with NW (Figure S1-A1) and of the OB[St+] patients compared with the OB[St−] patients (Figure S1-A2) is reported in supplementary Figure S1. 

**Figure 2:**
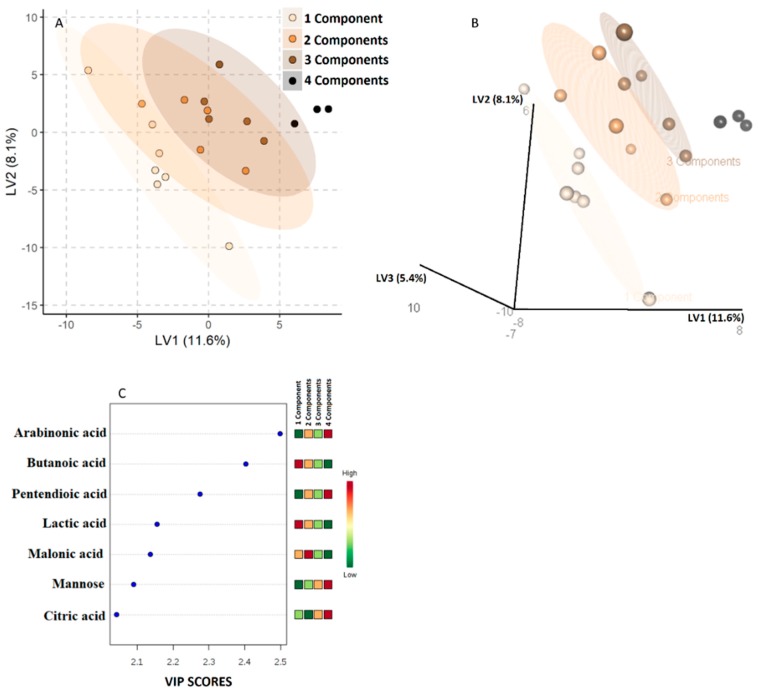
Partial least squares discriminant analysis (PLS-DA) model to discriminate obese children according to the number of Metabolic Syndrome (MetS) components. The explained variance of each component is shown on the corresponding axis. In panels **A** and **B**, the color darkness progression denotes the MetS components increase. The seven metabolites with a variable important in projection score (VIP-score) higher than 2 are shown in Panel **C**.

The levels of valine, mannose, acetopyruvic acid, palmitic acid, triethylene glycol, gluconic acid, citric acid, scyllo-inositol, deoxyglucose, psicopyranose, myo-inositol and cycloserine were higher in OB patients (Figure 2B1). Conversely, the levels of 1,2,3-butanetriol, 2-oxovaleric acid, 2-palmitoylglycerol, Di-n-octyl phthalate, itaconic acid, methyl galactoside, stearic acid, 2-piperidinone, maltose, 2-deoxy-d-ribose, pentane dioic acid, glycerol, pentitol, glyceric acid, methyl maleic acid, 2-deoxypentofuranose, β-hydroxy pyruvic acid, 2-hydroxy- methylcyclopentanol, and L-serine were higher in NW patients (Figure S1-B1). OB[St+] patients had higher levels of d-glucuronic acid γ-lactone, 2′-deoxyribolactone, 2-hydroxyisocaproic acid, pyroglutamic acid, and propanoic acid. Instead, OB[St−] patients had higher levels of butanoic acid, maltose, thiamine, glucopyranose, 2-hydroxybutyric acid, and mannose (Figure S1-B).

Figure 2 represents the PLS-DA model regarding the aggregation of saliva samples by the number of MetS components. 

A clear-cut class separation was achieved, following the increase in the number of MetS components (Figure 2A,B). The metabolites with a VIP-score > 2 were as follows: arabinoic, butanoic, pentendioic, lactic, malonic and citric acid and mannose (Figure 2C).

**Figure 3:**
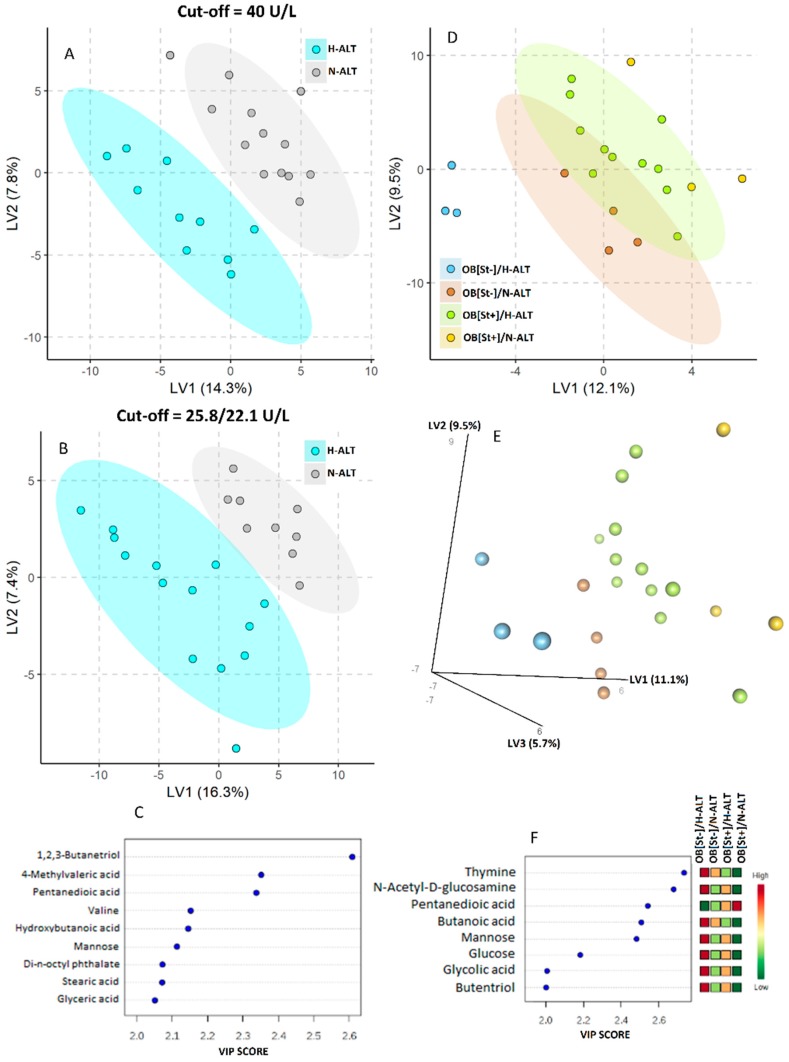
Partial least squares discriminant analysis (PLS-DA) model to discriminate children according to the presence/absence of hypertransaminasemia. Panel **A**: Serum Alanine transaminase (ALT) > 40 U/L was considered as hypertransaminasemia for both boys and girls. The explained variance of each component is shown on the corresponding axis. Panel **B**. Serum ALT > 25.8 U/L for boys and 22.1 U/L for girls was considered as hypertransaminasemia. In panels **A** and **B**, the cyan ellipse contains children with ALT > cut off values, while gray circles represent the children with serum ALT lower than cut off values. The nine metabolites with a VIP-score higher than 2 are shown in Panel **C**. PLS-DA shown in Panels **D/E** cumulates information on the status of both hepatic steatosis and transaminases values with respective variable important in projection scores (VIP-scores) shown in Panel **F**.

Obese patients were also aggregated considering the serum ALT concentration. Figure 3A reports on the PLS-DA model when the serum ALT level higher than 40 mg/mL was considered hypertransaminasemia. Nine metabolites (butentriol, methyl valeric acid, pentanedioic acid, valine, hydroxy butanoic acid, mannose, di-n-octyl-phthalate and stearic and glyceric acid) showed a VIP-score higher than 2 (Figure 3C).

When the serum ALT level >25.8U/L for boys and 22.1 U/L for girls were considered hypertransaminasemia [24], the PLS-DA model remained discriminant (panel 3B), and the metabolites showing a VIP score >2 remained unchanged (panel 3C). PLS-DA shown in Panel 3D/E cumulates information on the status of both hepatic steatosis and transaminase values with respective VIP-scores shown in Panel F.

**Figure 4:**
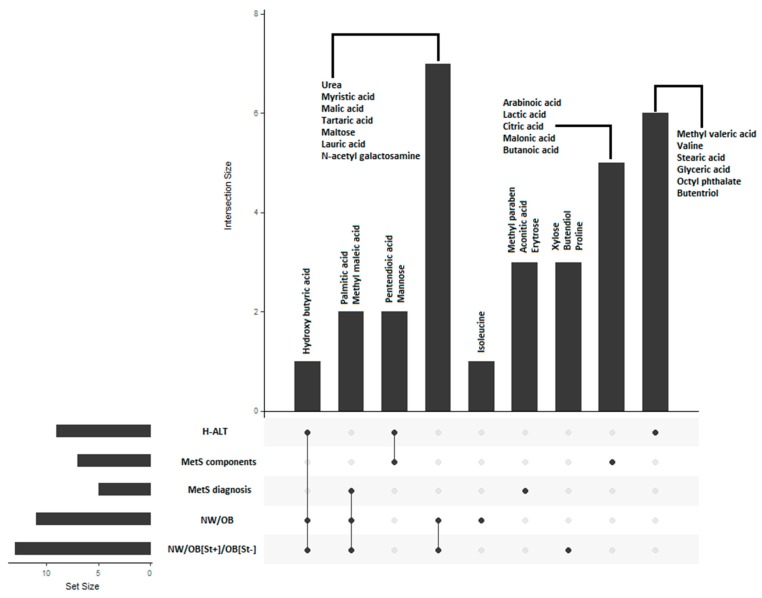
UpSet representation of the metabolites selected in the different classification models. H-ALT: Hypertransaminasemia; MetS: Metabolic Syndrome; NW: normal weight, OB: obese, [St]: hepatic steatosis.

Figure 4 illustrates the UpSet [37] representation summarizing the selected metabolites in several classifications and the relationships between sets.

**Figure 5:**
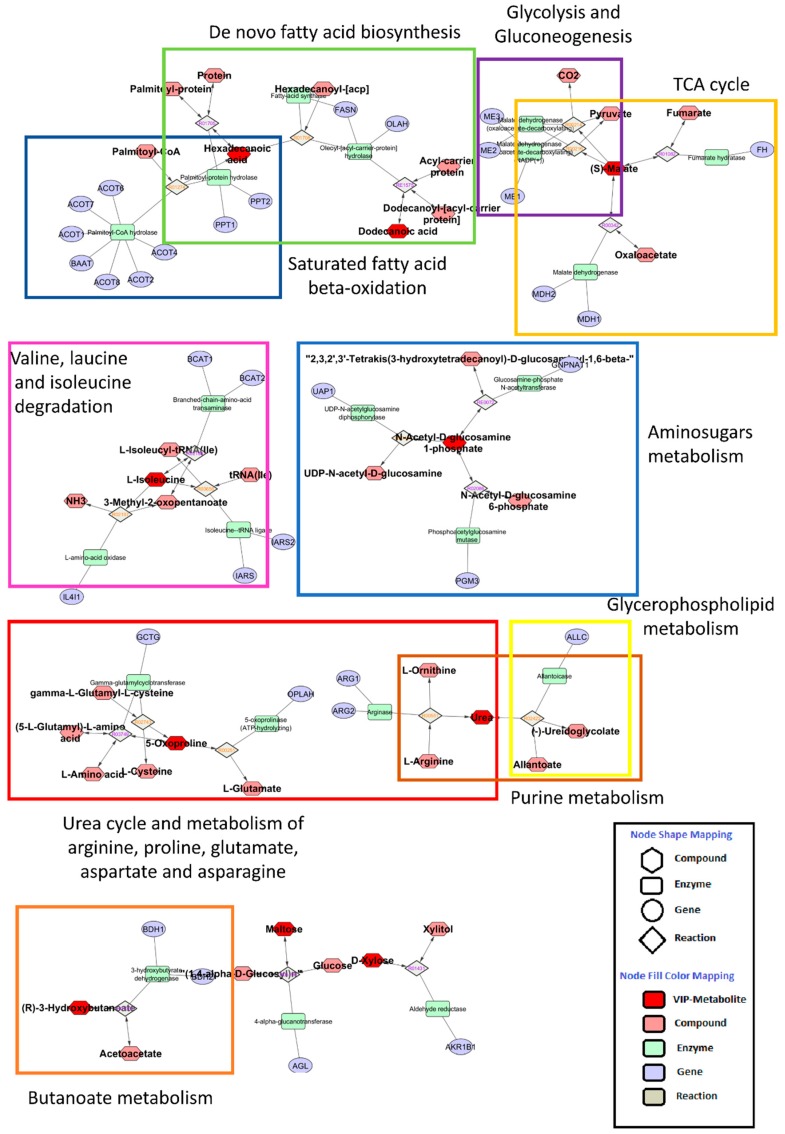
Metabolic systems map summarizing the shortest route that may explain the interactions among the metabolites with a variable important in projection scores higher than 2. There is a clear interplay of several pathways involving: de novo fatty acid biosynthesis; saturated fatty acid beta-oxidation; butanoate metabolism; glycolysis and gluconeogenesis; tricarboxylic acid cycle (TCA); urea cycle and metabolism of proline, glutamate, aspartate and asparagine; valine, and isoleucine (branched chain amino acids) degradation; aminosugars metabolism; purine metabolism; glycerophospholipid metabolism.

Overall, as shown in the metabolic systemic map (Figure 5), there is a definite interplay of several pathways involving the following processes: de novo fatty acid biosynthesis; saturated fatty acid beta-oxidation; butanoate metabolism; glycolysis and gluconeogenesis; tricarboxylic acid cycle; urea cycle and metabolism of proline, glutamate, aspartate and asparagine; valine, leucine and isoleucine (BCCA) degradation; amino sugar metabolism; purine metabolism; and glycerophospholipid metabolism.

## 4. Discussion

As in a few other conditions (pediatric celiac disease [38], mild cognitive impairment [2], sport performance/fatigue [3,39], T2D [5,40]/T1D [41], and some neurological conditions [42]), our study shows that salivary metabolomics may represent a useful tool to obtain additional pathomechanistic information and serve as a possible clue to individuate novel disease diagnostic biomarkers data also in pediatric obesity. From our results, overall it appears that several salivary metabolites and metabolic pathways contribute to a complex metabolic fingerprint of obesity, obesity-related NAFLD and obesity-related MetS. Some of these metabolites were easily predictable based on obesity pathophysiology whereas others were not.

In line with blood and urinary metabolomic results obtained by others [43,44,45], the BCAAs valine and isoleucine were among the AAs more prevalently involved in the obesity-deranged pathways, but they did not appear to accurately reflect specific hepatic [43] or metabolic [44,45] involvement. The network of salivary molecules separating the lean and obese groups in obese individuals (independently from having or not MetS/NAFLD comorbidities) was also notably characterized by higher levels of two saturated fatty acids, palmitic acid and myristic acid, which tended to be prevalent in those with steatosis. Interestingly, this finding is in line with recently reported data suggesting that elevated total serum ceramide, as well as specific concentrations of myristic, palmitic, palmitoleic, stearic, oleic, behenic and lignoceric ceramide, with insulin resistance and play a potential role in the development of NAFLD in obese children [46]. The correlation of the lipid profile with glucose and insulin levels has been reported to probably mirror a still preserved ability to adapt to a caloric challenge compared with metabolically unhealthy individuals [47,48], in line with recent suggestions that propose a fatty acid profile is a useful tool to explain part of the heterogeneity between abdominal obesity and MetS [11,48,49]. Others have reported that, in addition to palmitic and stearic acid, other FAs are deranged and that increased activity of C16 Δ9-desaturase and C18 Δ9-desaturase in parallel with decreased Δ5-desaturase activity may be a causative factor in disturbed fatty acid metabolism [50]. In line with recent mouse model studies [51] where chronic oral administration of myristic acid improved hyperglycemia by decreasing insulin-responsive glucose levels and reducing body weight, myristic acid in our enrichment pathway is a fatty acid that appears to be associated with obesity but not with MetS. Finally, patients with fatty liver had higher levels of salivary pyroglutamic acid, a metabolite that has recently been proposed as a possible diagnostic biomarker for more severe liver disease [52].

Even more interestingly, as seen also by others in blood [12], PLS-DA showed that the salivary metabolic profiles could correctly identify children with a fewer number of MetS criteria than those who displayed more. This suggests that metabolic profiles can stratify MetS subpopulations, therefore, paving the way for their utilization for both early disease diagnosis and monitoring in those with MetS. This appears particularly relevant as in a recent Clinical Report, the American Academy of Pediatrics (AAP) Committee on Nutrition [53] acknowledged that although several attempts have been made to define MetS in the pediatric population, the construct at this age is difficult to define and has unclear implications for clinical care. For this reason, the Committee focused on the importance of (a) screening for and treating each individual risk factor component of MetS and (b) increasing awareness of comorbid conditions including NAFLD to be addressed and referred to specialists, as needed.

### Study Limitations and Strengths

Our findings should be considered in the context of several study limitations, including a relatively small sample size, methodological flaws, and the lack of liver biopsy and prospective data during follow-up. First, our sample size was somewhat limited, and we may have had insufficient power to detect significant associations, particularly for stratified analyses. Larger series with patient follow-up are needed to confirm the preliminary results of our pilot study. Second, our findings related to VIP metabolites should be interpreted with caution given that these were obtained on only one saliva sample for each of the participant children. Although saliva was revealed to be a reliable biofluid for metabolomics studies [17], neurological disorder [42], and T1D [41], the likely risks of poor reproducibility persist. In fact, possible, differences among unstimulated, stimulated (e.g., obtained with oral movements such as gentle mastication), and pure parotid saliva exist [54,55]. Third, ultrasound may be insensitive compared with biopsy or magnetic resonance imaging (MRI). Nevertheless, it is the reference test for use in pediatric clinical practice. Furthermore, liver biopsy cannot be considered a screening procedure because it is invasive, not riskless and not exempt from possible sampling errors. As a non-invasive alternative to assess hepatic steatosis, US is repeatable because it does not require sedation or the delivery of ionizing radiation [1,56]. Although it is the less robust of the numerous imaging options [57], methodological progress has shown good diagnostic specificity and sensitivity, especially if the steatosis involves at least 20% of the hepatocytes [58]. Overall, these limitations do not allow us to draw definite conclusions but strongly suggest the viability of such an approach. These limitations, however, are balanced by several important strengths, including a full auxological and biochemical characterization of our subjects’ cohort that allowed us to build several classification models on the same group of patients and delineate the metabolite/metabolic pathways. Moreover, this represents the first study to show the potential usefulness of saliva to define a metabolomic signature of pediatric obesity and related hepato-metabolic comorbidities.

## 5. Conclusions

Using the saliva of children affected by obesity, we showed a definite interplay of several metabolic pathways with possible specific patterns capable of sorting fatty liver and MetS. The involved metabolic processes include the following: de novo fatty acid biosynthesis; saturated fatty acid beta-oxidation; butanoate metabolism; glycolysis and gluconeogenesis; tricarboxylic acid cycle; urea cycle; metabolism of proline, glutamate, aspartate and asparagine; valine, leucine and isoleucine (BCAA) degradation; aminosugar metabolism; purine metabolism; and glycerophospholipid metabolism. Overall, this information, along with that of other recent progress regarding the study of salivary simple analytes [4], trace elements [59], major adipocytokines [60,61], and specific microRNAs [62], reinforces the idea that saliva will soon represent a useful tool for deepening pathomechanismistic aspects, noninvasive diagnosis and monitoring of pediatric and adult individuals with obesity. The early and non-invasive detection of incipient MetS/fatty liver in childhood through salivary metabolomics as described here, therefore, appears as a promising helpful tool to prevent further health hepato-metabolic and cardiovascular complications in adulthood, and ultimately serves to minimize their related global socioeconomic burden. 

## Figures and Tables

**Table 1:** Characteristics of the study population.

Anthropometric and Laboratory Parameters	Controls (*n* = 18)	Obese with Steatosis (*n* = 15)	Obese without Steatosis (*n* = 8)	All Obese (*n* = 23)
Gender (M/F)	13/5	10/5	4/4	14/9
Age (years)	10.53 ± 2.57	12.48 ± 2.77 *	12.51 ± 2.79 *	12.49 ± 2.71 *
Weight (kg)	37.42 ± 11.26	79.99 ± 28.76 *	71.9 ± 17.31 *	77.18 ± 25.24 *
Height (cm)	140.17 ± 15.17	153.41 ± 19.27 *	157.45 ± 11.97 *	154.52 ± 16.88 *
BMI (kg/cm^2^)	18.52 ± 2.92	32.80 ± 6.94 *	28.93 ± 5.58 *	31.45 ± 6.65 *
BMI percentile	23.75 ± 34.25	95.14 ± 0.53 *	95.67 ± 1.03 *	95.40 ± 1.05 *
Waist circumference (cm)	61.14 ± 7.11	93.27 ± 12.68 *	86.00 ± 14.53 *	90.74 ± 13.49 *
WC percentile	65.85 ± 24.58	94.98 ± 0.97 *	94.38 ± 1.77 *	94.78 ± 1.04 *
Cm WC > 95th percentile	0	21.03 ± 10.57 *	14.00 ± 10.99 *	18.59 ± 11.01 *
WtHR	0.43 ± 0.03	0.61 ± 0.05 *	0.55 ± 0.08 *	0.59 ± 0.07 *
Neck circumference (cm)	27.67 ± 2.41	36.05 ± 4.33 *	34.69 ± 4.08 *	35.58 ± 4.20 *
NC percentile	44.12 ± 33.22	95.57 ± 5.35 *	92.61 ± 3.15	94.09 ± 4.26 *
Cm NC > 95th percentile	0	3.71 ± 2.77 *	2.41 ± 2.75 *	3.26 ± 2.77 *
SBP (mmHg)	95.98 ± 11.95	127.47 ± 8.95 *	125.63 ± 20.23 *	126.83 ± 13.49 *
SBP percentile	50.00 ± 0	86.93 ± 19.36 *	83.50 ± 20.96 *	85.74 ± 19.52 *
DBP (mmHg)	55.00 ± 10.77	61.53 ± 10.42 *	60.75 ± 11.70 *	61.26 ± 10.62 *
DBP percentile	50.00 ± 0	56.00 ± 15.83 *	55.00 ± 14.14 *	55.65 ± 14.95 *
ALT (U/L)	17.33 ± 4.31	50.17 ± 28.75 *	34.50 ± 37.74 *	44.72 ± 32.21 *
AST (U/L)	24.72 ± 4.87	46.19 ± 28.58 *	19.75 ± 5.85	37.00 ± 26.39 *
Total cholesterol (mg/dL)	148.78 ± 16.38	158.17 ± 21.91 *	162.00 ± 24.20 *	159.50 ± 22.26 *
HDL (mg/dL)	56.94 ± 14.45	45.07 ± 10.21 *	48.00 ± 5.50 *	46.09 ± 8.83 *
Triglyceride (mg/dL)	Not available	90.59 ± 26.97	138.63 ± 91.90	107.30 ± 60.80
Blood glucose (mg/dL)	83.17 ± 6.61	88.59 ± 10.36 *	90.00 ± 10.34 *	89.08 ± 10.14 *
Salivary glucose (µM)	3338.36 ± 1274.73	3167.86 ± 1192.75	2647.09 ± 1227.77	2986.70 ± 1203.86
Blood insulin (U/L)	10.27 ± 5.22	24.24 ± 10.95 *	19.60 ± 6.63 *	22.62 ± 9.77 *
Salivary insulin (nM)	5.79 ± 2.85	20.89 ± 8.69 *	17.26 ± 6.37 *	19.60 ± 8.00 *
Blood HOMA-IR	2.01 ± 1.16	5.34 ± 2.60 *	4.11 ± 2.16 *	4.91 ± 2.48 *
Salivary HOMA-IR	119.7 ± 73.99	401.81 ± 231.17 *	278.79 ± 162.48 *	358.20 ± 215.35 *
Blood uric acid (mg/dL)	4.04 ± 0.76	5.06 ± 1.23 *	4.42 ± 0.92 *	4.84 ± 1.15 *
Salivary uric acid (µM)	143.46 ± 4.53	157.29 ± 13.04 *	156.45 ± 15.31 *	157.00 ± 13.53 *

Abbreviations = ALT: alanine transaminase; AST: aspartate transaminase; BMI: Body Mass Index; DBP: diastolic blood pressure; HDL: high density lipoproteins; HOMA-IR: Homeostasis Assessment Model—Insulin Resistance WC: waist circumference; NC: neck circumference; SBP: systolic blood pressure; WtHR: Waist to Height Ratio; * *p* value < 0.05 compared to controls.

**Table 2:** Metabolic Syndrome components in obese patients with and without hepatic steatosis.

	Number (%) of Obese Patients with Hepatic Steatosis	Number (%) of Obese Patients without Hepatic Steatosis	Total (%)
Sample size	15(65%)	8(35%)	23(100%)
Waist circumference >90th percentile	15(65%)	7(30%)	22(95%)
Glucose blood levels >100 mg/dL	4(17%)	2(9%)	6(26%)
Blood pressure >95th percentile	10(43%)	4(17%)	14(60%)
HDL <40 mg/dL	3(13%)	0(0%)	3(13%)
TG >150 mg/dL	2(9%)	3(13%)	5(22%)
HOMA-IR > 3	13(57%)	5(22%)	18(79%)
Numbers of patients fulfilling MetS Criteria: (WC > 90th percentile and more than two out of four other criteria)	7(30%)	3(13%)	10(43%)

Abbreviations = HDL: high density lipoproteins; HOMA-IR: Homeostasis Assessment Model – Insulin Resistance; MetS: Metabolic Syndrome; TG: Triglycerides; WC: waist circumference

**Table 3:** Variables important in projection (VIP) metabolites fold changes in patients versus controls’ saliva.

VIP	NW (*n* = 18) ^a^	OB[St−] (*n* = 15)	OB[St+] (*n* = 8)	*p*-Value ^b^	MetS− (*n* = 38) ^a^	MetS+ (*n* = 3)	*p*-Value ^c^
Hydroxy butyric acid	0.00697	−0.14	−0.62 *	NS	0.00622	−1.02	NS
Palmitic acid ^d^	0.00088	4.46 ***	8.06 **	NS	0.00398	−0.74	NS
Myristic acid	0.00092	3.71 **	7.58 *	NS	0.00375	−0.66	NS
Lauric acid	0.00061	−7.21 **	−3.35	NS	0.00267	0.73	NS
Urea	0.00093	4.15 **	7.65 **	NS	0.00404	−0.71	NS
*N*-acetyl galactosamine	0.00088	3.72 **	7.60 *	NS	0.00375	−0.66	NS
Malic acid	0.17825	−0.98	−0.98	NS	0.09066	0.96	NS
Methyl maleic acid	0.01375	−0.72	−0.24	NS	0.01164	0.81	NS
Maltose	0.07047	−0.54	−0.25	NS	0.05846	0.24	NS
Xylose	0.00864	−0.62	−0.34	NS	0.00681	0.27	NS
Butanediol	0.00070	−6.16 **	−2.79	NS	0.00272	0.34	NS
Proline	0.00999	−0.56	−0.25	NS	0.00752	−1.02	NS
Tartaric acid	0.06401	0.52	0.40	NS	0.04729	−0.40	NS

* indicates a *p*-value < 0.05 compared to NW, ** indicates a *p*-value < 0.01 compared to NW, *** indicates a *p*-value < 0.001 compared to NW, NS indicates a *p*-value > 0.05. **^a^** Normalized chromatographic peak area; **^b^** *p*-values of OB[St+]/OB[St−] comparison; **^c^** *p*-values of MetS−/MetS+ comparison; **^d^** Metabolite selected by both PLS-DA models. Abbreviations: MetS−: No metabolic syndrome diagnosis; MetS+: Diagnosis of metabolic syndrome; NW: Normal Weight; OB[St+]: Obese without steatosis; OB[St+]: Obese with Steatosis; PLS-DA: Partial Least Squares Discriminant Analysis; VIP: Variable Important in Projections

## Supplementary Materials

The following are available online at http://www.mdpi.com/2072-6643/11/2/274/s1, Figure S1: Panels (A) show the selected metabolites with fold change (FC) values <-1 or >+1 with a simultaneous *p*-value < 0.05 (red dot). (A1) Normal weight (NW) versus Obese (OB) metabolite. (A2) Steatosis obese patients (OB[St+]) versus non-steatosis obese patients (OB[St−]). FC of the selected metabolites are shown in the corresponding panel (B1 and B2).
