# Supplementary material for: Metabolomic Salivary Signature of Pediatric Obesity Related Liver Disease and Metabolic Syndrome
Source: Nutrients. 2019 Jan 26;11(2):274. doi: 10.3390/nu11020274 (PMC6412994; doi:10.3390/nu11020274)
Supplement: Supplementary file 1 [file nutrients-11-00274-s001.pdf]

A1

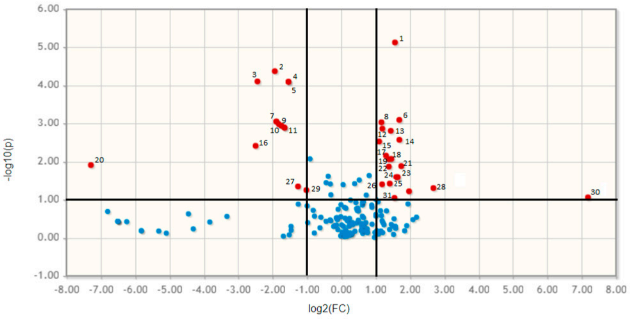

1. Glycerol
2. Palmitic acid
3. Acetopyruvic acid
4. Deoxyglucose
5. D-Psicopyranose
6. Methylmaleic acid
7. Triethylene glycol
8. 2-Palmitoylglycerol
9. D-Gluconic acid
10. Citric acid
11. Scyllo-inositol
12. Di-n-octyl phthalate
13. Maltose
14. 2-Deoxypentofuranose
15. 2-Oxovaleric acid
16. D-Mannose
17. Itaconic acid
18. 2-Deoxy-D-ribose
19. Stearic acid
20. L-Valine
21. Hydroxypyruvic acid
22. Methyl galactoside
23. Glyceric acid
24. Pentitol
25. 2-Piperidinone
26. 1,2,3-Butanetriol
27. Myo-inositol
28. 2-Hydroxymethylcyclopentanol
29. Cycloserine
30. L-Serine
31. Pentanedioic acid

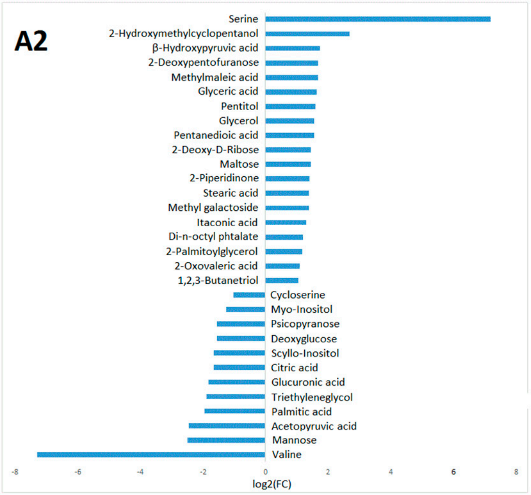

B1

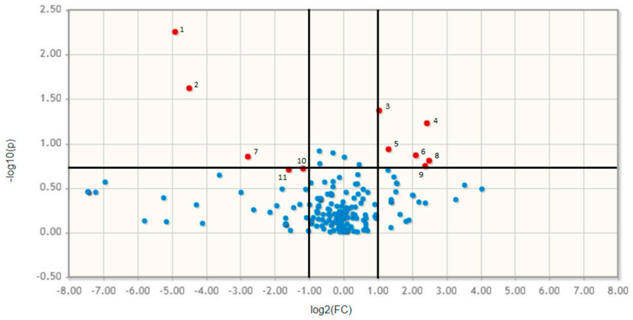

1. D-Glucuronic acid-γ-lactone
2. 2'-Deoxyribolactone
3. Butanoic acid
4. 2-Hydroxybutyric acid
5. Maltose
6. Thiamine
7. 2-Hydroxyisocaproic acid
8. Mannose
9. Glucopyranose
10. Propanoic acid
11. Pyroglutamic acid

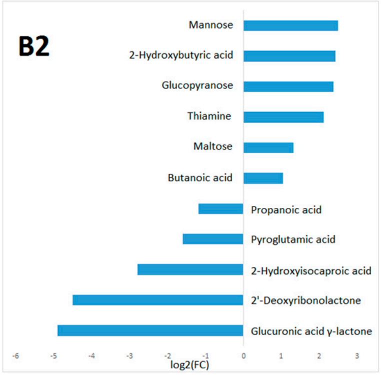

Figure S1. Panels (**A**) show the selected metabolites with fold change (FC) values <-1 or >+1 with a simultaneous p-value<0.05 (red dot). (**A1**) Normal weight (NW) versus Obese (OB) metabolite. (**A2**) Steatosis obese patients OB[St+] versus non-steatosis obese patients OB[St-]. FC of the selected metabolites are shown in the corresponding panel (**B1** and **B2**).
